# Silk Sericin Protein Materials: Characteristics and Applications in Food-Sector Industries

**DOI:** 10.3390/ijms24054951

**Published:** 2023-03-03

**Authors:** Su-Jin Seo, Gitishree Das, Han-Seung Shin, Jayanta Kumar Patra

**Affiliations:** 1 Department of Food Science and Biotechnology, Dongguk University-Seoul, Goyang-si 10326, Republic of Korea; 2 Research Institute of Integrative Life Sciences, Dongguk University-Seoul, Goyang-si 10326, Republic of Korea

**Keywords:** sericin, food packaging material, functional foods, antioxidant, anticancer, anti-tyrosinase

## Abstract

There is growing concern about the use of plastic in packaging for food materials, as this results in increased plastic waste materials in the environment. To counter this, alternative sources of packaging materials that are natural and based on eco-friendly materials and proteins have been widely investigated for their potential application in food packaging and other industries of the food sector. Sericin, a silk protein that is usually discarded in large quantities by the sericulture and textile industries during the degumming process of manufacturing silk from silk cocoons, can be explored for its application in food packaging and in other food sectors as a functional food and component of food items. Hence, its repurposing can result in reduced economic costs and environmental waste. Sericin extracted from silk cocoon possesses several useful amino acids, such as aspartic acid, glycine, and serine. Likewise, sericin is strongly hydrophilic, a property that confers effective biological and biocompatible characteristics, including antibacterial, antioxidant, anticancer, and anti-tyrosinase properties. When used in combination with other biomaterials, sericin has proved to be effective in the manufacture of films or coating or packaging materials. In this review, the characteristics of sericin materials and their potential application in food-sector industries are discussed in detail.

## 1. Introduction

**Figure 1:**
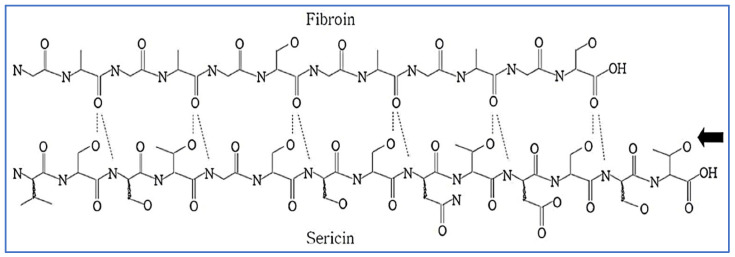
Structure of silk showing the intermolecular hydrogen bonds between the fibroin and sericin. Reproduced with permission from Lee et al. [11], 2004, John Wiley and Sons (originally Scheme 1).

Silk fiber, which is an essential component of the textile or sericulture industry, is mainly produced by a number of silkworms belonging to the families Bombycidae, Lasiocampidae, and Saturniidae, which include a few spiders [1,2,3,4]. The most commonly used silk is from silk cocoons made by the silkworm *Bombyx mori* [1]. As per the data statistics, published by the International Sericulture commission, in the year 2019 and 2020, 1,09,111.10 and 91,771.00 MT of silk cocoons respectively was produced globally [5]. Silk fibers are mainly composed of two types of protein, fibroin and sericin (Figure 1). As an adhesive substance, sericin surrounds the exterior of fibroin, which lies at the center of silk fibers [6]. In the degumming process, sericin is usually separated from fibroin and discarded, and only the fibroin is used as silk [3,6]. Sericin is detached from fibroin by the silk industry in order to improve the smoothness, luster, lightness, and dyeability of the fibers [3,7]. As sericin is a major component of raw silk, it has been estimated that out of 4.0 lakh tons of dry cocoons produced worldwide, around 50,000 tons of sericin are usually discarded as waste in sewage, representing an environmental hazard [3,8,9,10]. 

**Figure 2:**
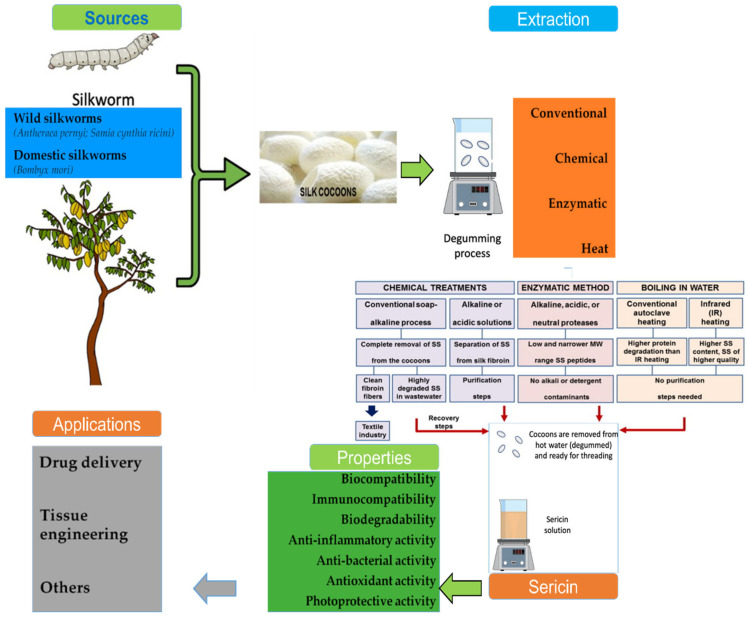
Graphical presentation of the source, extraction process, characteristics, and applications of sericin. Adopted from Silva et al. [10] under the terms and conditions of the Creative Commons Attribution (CC BY) license (https://creativecommons.org/licenses/by/4.0/), 2022, Licensee MDPI, Basel, Switzerland, (Originally Figure 1); from Das et al. [12] under a Creative Commons Attribution 4.0 International License, 2021, Springer Nature (originally Figure 2); and from Lamboni et al. [3], with permission, 2015, Elsevier (originally Figure 1).

It is reported that sericin has a number of useful applications, starting from useful food elements to applications in the cosmetic and medical fields [6,10,12]. In addition, sericin’s biochemical and biophysical characteristics vary depending on the process used for extraction. Sericin has been extracted using a variety of methods, such as hot water, acidic, citric acid solution, urea, and sodium carbonate solution; however, the degumming is the method most commonly adopted by industries (Figure 2). The different extraction methods for silk might result in varying molecular weights, amino acid content, conformation, zeta power, and particle size, as well as different physical–chemical properties and biological activities of sericin [13,14]. Consequently, different factors, such as pH, temperature, processing time, and chemical reagents, have a major impact on sericin content [15,16].

Sericin contains 18 amino acids, including positively and negatively charged, aromatic, polar, and nonpolar amino acids [15,17]. Moreover, these properties of sericin have made it useful for a variety of purposes and particularly in food-sector industries. The polar chemical groups of amino acid side chains, such as hydroxyl and carbonyl, allow for crosslinking copolymerization and the formation of blends with other polymers [12,18]. Sericin protein’s polar chemical groups possess antioxidant, antibacterial, and anti-tyrosinase properties, along with anti-inflammatory, antitumor, and anti-GST activity (Figure 2) [3,10,12,19]. Serine is found in the largest proportion (40.51%) of the polar amino acid side chains present in sericin [19]. In addition, sericin also contains low-molecular-weight (<20 kDa) and high-molecular-weight (>20 kDa) amino acids, each of which are useful in various applications [12,18]. Those of low molecular weight can be used as biomaterials, health, cosmetics productions, and medications, while those of high molecular weight are usually used as biomaterials, membranes, hydrogels, and compound polymers [15,19]. A variety of applications of sericin, including in cosmetics, pharmaceutics, food, and textiles, have also been reported [13].

Because of its unique characteristics, such as biocompatibility with hydroxyapatite and other biocompatible and biodegradable materials, a number of applications and uses of sericin have been proposed [18,20]. However, it has been discarded in large amounts by the silk industry during the manufacture of silk, and very few studies have reported or been recently conducted on reuse of this waste material [3,13]. Therefore, the utilization of sericin in numerous ways, including in applications of the food sector, is crucial to develop its potential environmental, social, and economic benefits as a high-value-added material [13]. 

Currently, scientists around the world are searching for alternative sources of coating materials for use as food packaging in food industries [21]. In this scenario, the low-cost edible coatings could serve as an alternative packaging/coating materials with semipermeable properties, providing a barrier against oxygen, carbon dioxide gasses, and moisture content that could decrease rates of respiration, water loss, and oxidation [22]. A number of natural biomaterials, including proteins, polysaccharides, lipids, and resins, individually or in combinations, are commonly utilized as edible coating materials around the world [22]. However, these are sometimes expensive and therefore not commercially viable [21]. To make them commercially feasible, safe, and cost-effective, attention is focused on the utilization of inexpensive and sustainable resources, and for this purpose, the use of widely available and industrially wasted protein materials such as sericin has been explored [21,23]. It is reported that sericin, which has no fat, can serve as a promising and safe alternative material for manufacturing lipid-free coatings, such as polyglycerol–polystearate-emulsion-based coatings [24]. A few studies by both Chimvaree et al. [25] and the Thongsook and Tiyaboonchai group [26] reported that sericin coating on fruits such as apples and mangoes and vegetables such as mushrooms resulted in decreases in weight loss and browning and polyphenol oxidase activity by around 40% [25,26]. In another report, it was shown that the application of thin sericin coating layers on bananas and strawberries is highly effective for preservation [27].

Sericin, as industrial waste from the textile and silk industry, can serve as a sustainable and cheaper source of protein for use as a fat-free edible coating material [21]. In addition, it has been reported to be effective in reducing the weight loss of food material during storage, together with enhancing the color and texture of fruits and inhibiting the polyphenol oxidase enzyme activity, thereby maintaining the freshness of fruits [26]. In earlier reports, it is clear that, in use, sericin-based coating materials have advantages over other biomaterial (lipid)-based coating materials, such as minerals, synthetic waxes, and animal and vegetable waxes, and they are also considered to be a generally recognized safe material if used in very thin layers [1,6]. Furthermore, the film-forming ability of sericin has been reported to be enhanced by the combination of other polysaccharides, such as glucomannan [23]. Consequently, there is enormous opportunity for sericin-based coating materials and films through combination with other biomaterials, such as proteins, polysaccharides, and gums, which can be highly effective in enhancing the functional properties of edible films and coats by imparting beneficial properties, such as antibacterial, antioxidant, and tunable water-barrier properties [28]. Considering the vast potential of sericin globular protein and its superiority among other biomaterials, we describe the notable characteristics of sericin in the current review and explore its potential applications in food-sector industries.

## 2. Sericin: Extraction Process and Characteristics

There are various methods for extracting sericin from silk cocoons, of which the conventional method of extraction using only hot water is very common. It has the advantage of allowing the use of the natural polymer, although the purity of sericin, in this case, is much lower than when using other extraction methods [29]. In addition, there is very limited information about the composition of the sericin extracted by the hot-water extraction method [30]. Another conventional sericin extraction method is the soap–alkaline degumming process using Marseille soap and chemicals such as a solution of sodium bicarbonate (Na_2_CO_3_) boiled at atmospheric pressure under a high temperature [10]. However, in this process, the recovery of sericin and its quality are very low, resulting in the loss of its functional properties [10]. Usually, the boiling process in extraction substantially affects the silk structure, and after prolonged periods of boiling, the molecular weight is lowered due to fragmentation of sericin, which results in the degradation of the amorphous regions, including of their hydrophilic properties [18]. Considering the deficiencies in the conventional extraction procedures, several other types of extraction methods are being developed for the extraction of sericin (Figure 2) [3]. These methods can be divided into three main groups: physical, enzymatic, and chemical (Table 1) [3]. 

In the case of enzymatic extraction procedures, the amino acids, DPPH radical-scavenging activity, and total essential amino acid value obtained for sericin are noteworthy because they are three times higher than those obtained by water-extraction methods [33]. Compared with the hot-water extraction process, the use of a protease enzyme in the sericin extraction process causes the breakage of peptide bonds in protein molecules, resulting in free amino acids and shorter peptides [33]. However, this approach has the limitation of being expensive to use for large quantities [33]. In the case of chemical extraction methods, sodium carbonate, urea–mercaptoethanol, urea, sodium chloride, etc., are used, but this process requires an additional step of purification to remove the chemicals, and it is also difficult to extract high-quality sericin [3]. In some cases, calcium chloride is used to recover high-purity sericin in the extraction process by separating sericin from surfactants [29]. Currently, extraction methods involving the use of sodium carbonate or other types of alkali medium are widely used at industrial scales [31].

## 3. Physicochemical Characteristics of Sericin

### 3.1. Amino Acid Composition

The amino acid content of sericin has always been the same; however, extraction methods have a slight influence on the proportion of amino acids [34]. The amino acid profile of sericin, as reported in different publications, is presented in Table 2. Most of the relevant publications have shown that, of all the amino acids present in sericin, serine is present in the highest proportion, at about 30% [18,19,29,34,35,36,37,38]. The next most abundantly represented amino acids in sericin, after serine, are aspartic acid and glycine [39]. Serine plays an important role in determining the functional and physiochemical properties of sericin due to its strong polar hydroxyl groups [36]. Aspartic acid and glycine are also important in terms of sericin function. Moreover, sericin also contains hydrophilic amino acids (around 70%), resulting in its good solubility and water permeation properties [39]. Furthermore, aromatic acids are found at much lower quantities, such as phenylalanine (6.6%) [39]. 

The yield of extracted sericin is enhanced when sericin is abundant in polar amino acids and is extracted under alkaline conditions [3]. Additionally, the sericin yield is dependent on the amount of ethanol added during the extraction process [3]. Increasing the amount of ethanol results in an increased amount of amino acids such as serine, threonine, and aspartic acid and a decreased amount of amino acids such as alanine, leucine, isoleucine, glutamic acid, phenylalanine, and histidine in the extracted sericin [39]. This confirms that a high concentration of ethanol in the extraction process increases the content of hydrophilic proteins and decreases the content of hydrophobic proteins in the extracted sericin [39]. Higher yields have also reported for using 75% ethanol rather than 90% ethanol [39].

The secondary structure of proteins includes β-sheet structures [40,41]. Sericin extracted using hot water has a lower quantity of β-sheet structure due to decreased hydrogen bonding between sericin and fibroin as well as reduced coupling [40,41]. Due to its insolubility in hot water, the sericin that is most closely bonded to fibroin exhibits a higher proportion of the β-sheet structure than other groups of sericin [41]. The process of extraction with Na_2_CO_3_ contributes to denaturation of the β-sheet structure in the secondary protein structure and facilitates water permeation inside the silk cocoon and, thereby, the removal of sericin [40,41]. However, sericin extracted using hot water contains the highest amount of sulfur-containing amino acids, such as methionine and cysteine, which facilitate formation of double-helical structures. These amino acids are highly associated with collagen production [34]. 

The high contents of serine and threonine can affect the antioxidant properties of sericin, and these hydroxyl amino acids act as a chelating agent for trace elements such as iron and copper [35]. Further, sericin’s polyphenol and flavonoid contents might also contribute to its antioxidant activity. Natural pigments, which accumulate in sericin, could be provided by silk cocoons and are typically flavonoids and carotenoids. Due to their biological antioxidant and anti-tyrosinase characteristics, the presence of these pigments in sericin may be essential for preventing microbial and UV deterioration. [18,42]. Therefore, sericin is safe and could potentially be used as a natural component in food packaging [18].

In another study by Silva et al., the authors demonstrated how different extraction methods affect the amino acid and functional group composition of sericin [10]. Others have claimed that the extraction method does not influence the serine concentration, which remains the most abundant of the amino acids in sericin, but that the methionine content of sericin is higher when extracted using the heat treatment method than when using other methods [34]. Similarly, the amount of tyrosine content in sericin was significantly lower when extracted using the urea extraction method than when using other methods [34]. Details of the changes in amino acid composition of sericin extracted using different extraction processes are shown in Table 3. Furthermore, the contents of secondary metabolites and functional groups, such as phenols and flavonoids, present in sericin also vary with respect to the different extraction methods [43]. The total phenol content was found to be higher when sericin was extracted using the heat (hot water) method and lower when sericin was extracted with urea solution; similarly, acid-degraded sericin yields higher total flavonoid content than the alkali degradation method [43]. 

### 3.2. The Fourier-Transform Infrared Spectroscopy (FTIR) Analysis of Sericin

**Figure 3:**
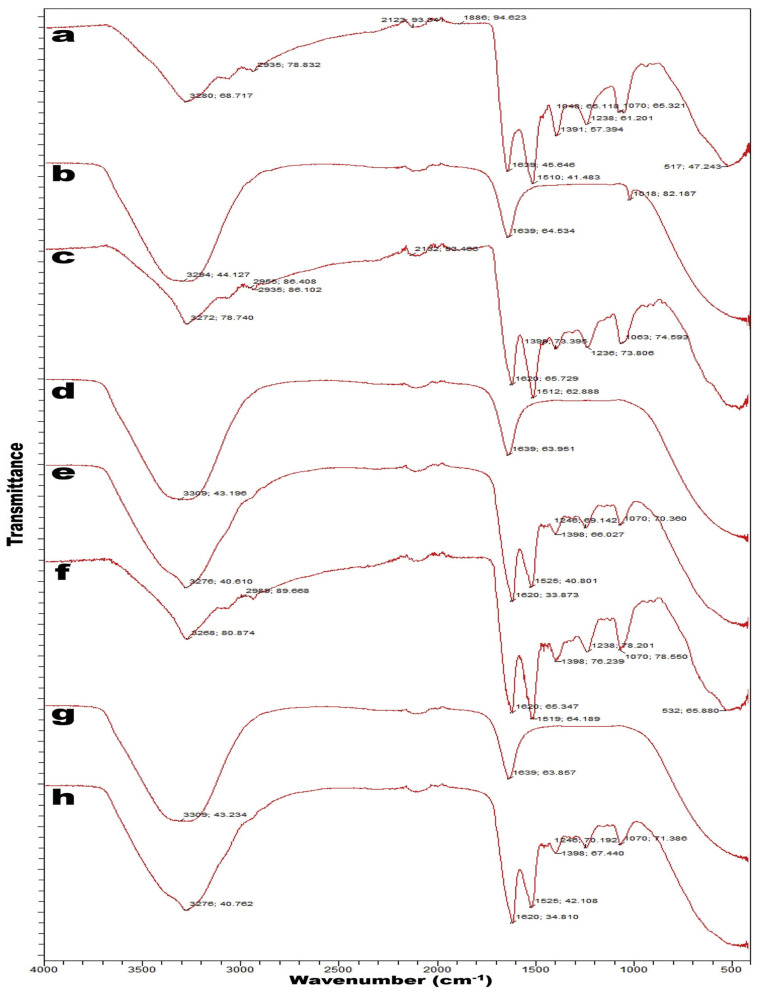
FTIR spectra of sericin: (a) sample in powder; (b) sample in solution; (c) sample in powder; (d) sample in powder aqueous supernatant; (e) sample in powder aqueous pellet; (f) sample in powder; (g) sample in powder aqueous supernatant; and (h) sample in powder aqueous pellet. Reproduced with permission from Rocha et al. [44], 2017, Elsevier (originally Figure 1).

The FTIR spectra of sericin are shown in Figure 3 [44]. As per Deepti Guptaa et al. [45], sericin showed three characteristics of vibration bands for amide I, II, and III at 1630–1650, 1540–1520, and 1270–1230 cm^−1^, respectively. In the primary amides, the 1650 and 1630 cm^−1^ bands confirm the presence of random coils and β-sheets, respectively [45]. The 1540 and 1520 cm^−1^ bands are the random coils and β-sheets, respectively, of secondary amides, whereas the 1270 and 1230 cm^−1^ bands indicate random coils and β-sheets, respectively, of tertiary amides [44,45]. The primary amides are made up of 70–85% C=O and 10–20% C-N groups. The secondary amides are more complex than the primary amides and correspond to bands in range from 1510 to 1580 cm^−1^ [46]. These results indicate the random coil conformation of sericin [47]. Sericin shows bands in FTIR spectra primarily between 1600 and 1700 cm^−1^; however, the signature peak for sericin is predominantly at 1400 cm^−1^ [45]. 

The absorption of primary amides, which is due to C=O stretching vibrations, is useful for determining the secondary structure of proteins [45]. C-N stretching combined with N-H plane bending is shifted in the range from 1240 to 1250 cm^−1^, corresponding to a change from the random coil to β-sheet structure [45]. Another signature peak of sericin in the FTIR spectra is found between 3284 and 3309 cm^−1^ due to the hydroxyl group (–OH) of the amino acid serine [44,47]. The major peak of sericin at 1639 cm^−1^ is due to the carboxyl group (C=O) of primary amides [44]. This results in the strongly hydrophilic character of primary amides [44]. Additionally, influences in N-H stretching vibrations are observed in peaks ranging from 3500 to 3000 cm^−1^, which overlap with peaks at 3600 to 3200 cm^−1^ due to O-H stretching [48]. The peaks at 2998 and 2951 cm^−1^ are associated with OCH_3_ and CH-aliphatic groups, respectively [49].

The molecular conformation and crystallization actions of sericin are depicted by the crystallinity index [50]. Considering that the 1645 cm^−1^ peak is related to random coil confirmation, it is attributed to the β-sheet crystallites in sericin. The absorption of this increases when the β-sheet crystallites of sericin are disrupted by heating at a high temperature, indicating an increase in random coil conformation [50,51]. Moreover, the crystallinity index values of sericin film decrease remarkably at high temperatures because the β-sheet crystallites are disrupted. Treatment with formic acid accelerates the disruption of β-sheet crystallites in sericin [50]. 

It has been reported that the molecular conformation of sericin, as examined by FTIR spectroscopy analysis, is highly essential because it affects the gelation of sericin solutions and thereby affects the mechanical properties of sericin films/coatings, which are essential in its food applications [50,52]. Park and Um also stated that different heat treatments during the preparation of sericin materials, such as gel, coats, films, fiber, etc., greatly affect the nature and structure of sericin, which can be studied by FTIR analysis [50]. The chemical aspects of sericin protein can be determined by FTIR analysis and allow their differentiation from other types of protein materials. In one study, Teramoto concluded that the strong absorption bands at around 1400 cm^−1^ are due to the side chains of serine residues that make up around 30% of the constituent amino acids of sericin, and this could be considered a signature for identifying sericin from other types of proteins [53]. The author also concluded that most of the sericin molecules are not oriented in the sericin fiber and that only a few oriented segments exist. However, in contrast to this, the absorption intensities of fibroin protein bands differ markedly for the parallel and the perpendicular spectra, which shows that the fibroin protein has a highly oriented structure and that the differences are due to the differences in the amino acid sequences between the two proteins [53,54].

### 3.3. Coloration

The color values corresponding to the sericin content of Tasar silk fibers were studied by Jena et al. [19]. The authors provided details for the three components of color, namely L*, a*, and b*. L* represents lightness and ranges from black (L* = 0) to white (L* = 100). Red/green and blue/yellow ranges are used for the a* and b* values, respectively, where the a* value ranges from −60 (greenness) to 60 (redness) and the b* value ranges from −60 (blueness) to 60 (yellowness) [19]. However, the characterization of the color values of sericin generated from *Bombyx mori* indicates the values are close to white (L* = 100), greenness (a* = −60), and yellowness (b* = −60) [55], which indicates high lightness and more green and yellow color intensities [55]. In another study, Park and Um stated that, depending on the type of sample, the color of sericin films can be changed by increasing the treatment temperature [50]. 

Real colorants in food items, such as in coating or films during food packaging, determine the overall look of items and may also be affected by how evenly they can be dispersed in a product without bleeding or precipitating out over time. Color stability can be affected by the moisture content and protein or fat composition of the packaging material, and it has been demonstrated that the coloration of sericin does not result in significant changes in their environmental parameters, as it is based on natural compounds that are more stable. In their study, Ma et al. showed that crosslinking the sericin material of naturally colored silk by using phytic acid could significantly decrease the water solubility and thus could improve the color fastness of the sample [56]. Based on this study, we can presume that the stability of sericin can be improved through the addition of other chemicals. Sericin has potential for application in the food sector, and its color might be an important factor because it could affect consumer preference for food after its packaging [57]. 

### 3.4. Molecular Weight Determination

Due to structural variations, the molecular weight of sericin macromolecules fluctuates in a range of about 10 to 400 kDa [15,19]. The molecular weight is affected by various types of extraction methods [24,58]. The molecular weight of sericin is 24,000 when extracted by hot water, 50,000 in the case of aqueous urea extraction, and around 3000–10,000 when applying an enzymatic extraction process [24]. Furthermore, another author presented that the chemical and biological properties of sericin may vary depending on the diversity in molecular weight distribution [34]. When sericin is extracted using an alkali, its molecular weight distribution is between 15 and 75 kDa, whereas when it is extracted using heat and acid, it is between 35 and 150 kDa [58]. In the case of sericin extraction with urea, the molecular weight ranges from 10 to >225 kDa, and for acid extraction, the range is from 50 to 150 kDa [34]. In addition, the amount of extracted sericin is much lower for acid and alkali than for urea [34]. Moreover, the use of high temperature and high pressure also affects the molecular weight range, which is between 25 and 150 kDa [34]. The range of molecular weight distribution is larger for sericin when extracted using Na_2_CO_3_ than when using hot water [40]. 

Sericin’s molecular weight shows a continuous distribution particularly between 14 and 97 kDa, and it is widely dispersed throughout this range [39]. Sericin of low molecular weight is primarily distributed in the 6–10 kDa range, owing to its capacity to degrade into lower-molecular-weight molecules during the degumming process [39]. The variation in the molecular weight distribution has a potential effect on the biological properties of sericin, such as the antibacterial and antioxidant activity, because there are at least 15 different polypeptide chains in the range from 20 to 220 kDa [34,39,58]. Consequently, the variety of degumming methods influences the molecular weight of sericin [58].

### 3.5. Elemental Composition of Sericin

Sericin is a macromolecule of hydrophilic amino acids and is composed of 18 amino acids. It has powerful polar groups, such as amino, carboxyl, and hydroxyl groups, that may participate in copolymerization, crosslink formation, and combination with other polymers [3]. The analysis of sericin’s elemental composition shows that it consists of 6% hydrogen, 46.5% carbon, 16.5% nitrogen, 31% oxygen, and 0.9% of sulfur [18,19]. The CN ratio is correlated with the solubility of hot water; therefore, when it is lower, the solubility in hot water is higher [19]. 

Sericin can be classified according to its molecular weight and solubility into three fraction groups, which, in order from highest to lowest, are sericin A, B, and C [18]. Sericin A, B, and C contain 17.2%, 16.8%, and 16.6% nitrogen, respectively [18]. Sericin A and B are also soluble in hot water, while sericin C is insoluble in hot water because it is close to fibroin. The amino acid composition differs for different sericin fraction groups, where sericin A and B commonly contain hydrolysis products of the amino acids alanine, arginine, isoleucine, leucine, glutamic acid, valine, and tyrosine in moderate concentrations; serine, aspartic acid, glycine, and threonine in high concentrations; and phenylalanine and lysine in relatively low concentrations [59]. Sericin B contains additional tryptophan [18]. The same amino acids as sericin B are present in sericin C; in addition, in the innermost layer, sulfur is present as an inorganic sulfide [59]. 

Sericin’s biochemical properties, such as its antibacterial activity, antioxidant properties, biocompatibility, and others, are an outcome of its content [18].

### 3.6. Absorption Spectra of Sericin 

**Figure 4:**
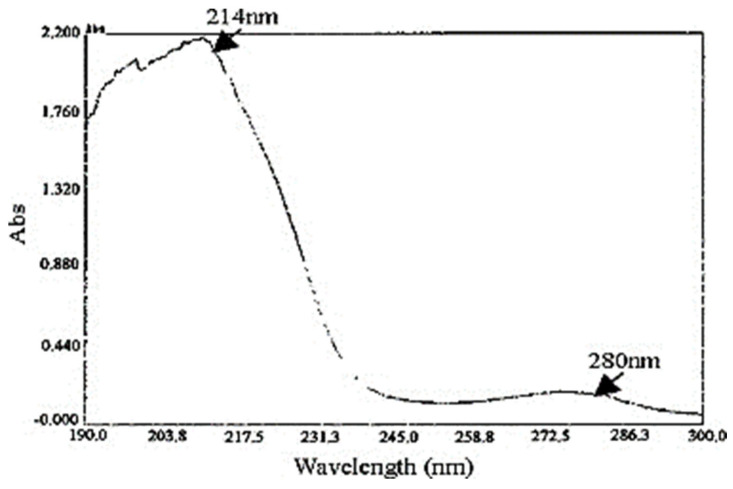
UV–Vis spectra of sericin. Reproduced with permission from Wu et al. [39], 2007, Elsevier (originally Figure 1).

It is stated that the peptide bonds and aromatic acids play a significant role in how strongly proteins absorb in the ultraviolet region [45]. Serine, which is found in high quantities in sericin, has strongly polar hydroxyl groups, which could affect the wavelength absorption of sericin [39]. In addition, tyrosine, tryptophan, and phenylalanine are the aromatic amino acids present in sericin [45]. The wavelength range from 190 to 300 nm was scanned in the UV–Vis absorbance spectra of sericin (Figure 4) [39]. The absorption maxima of sericin were obtained at 214 nm, which is due to peptide bonds, and other minor peaks that range from 274 to 280 nm are attributed to aromatic amino acids [19,39,60].

## 4. Application of Sericin in Food-Sector Industries

### 4.1. As a Food-Packaging and Food-Coating Material

There are reports that over 800 million people are starving around the world, and more than 40% of newly packaged food and locally farmed products are wasted. As 30% of food in retail outlets goes uneaten, extending the shelf life of the world’s food supply at room temperature by just one week would be extremely beneficial to the agriculture and food manufacturing sectors and drastically lower the size of the global waste stream (https://neo.life/2022/06/food-lasts-1-week-longer-with-this-edible-silk-coating/, accessed on 20 December 2022).

Natural food-packaging materials that are biodegradable for replacing synthetic polymers have been noted in response to the growing concern over plastic pollution [61]. Because synthetic polymers are nonbiodegradable and nonrenewable, they cause environmental pollution [62]. These issues of synthetic polymers can be reduced by using biodegradable polymers [62]. In addition, besides their biodegradable properties, biopolymers have other favorable functionalities, such as wide availability, renewability, and nontoxicity [63], and can be directly extracted from various natural sources [64]. Therefore, there are studies on various types of biopolymers regarding their characterization, properties, and the development of biopolymers in food packaging using raw materials, such as proteins, lipids, and polysaccharides to replace synthetic polymers [65,66]. Among them, protein-based materials can offer environmental compatibility in addition to enhancing food quality and shelf life [57]. 

Sericin was discovered to have good reactivity and a high range of biological functions, such as biodegradability, biocompatibility, antibacterial, and antioxidation [15]. A few patents and publications on the use of sericin in the food industry can be found [67]. These applications of sericin in the food sector are discussed here (Table 4). Sericin is reported to have certain limitations for use in food packaging due to its weak mechanical properties, since stimulated self-aggregation of sericin induces weak mechanical strength [68]. Moreover, its hydrophilicity nature makes it unsuitable for a watery environment, which is a significant drawback that prohibits its use in a variety of industries [69]. Fortunately, the lipid permeability, as well as other physical characteristics of sericin, such as the moisture content, light transmission, swelling, transparency, and solubility in films, are not affected [68]. Hence, extracted sericin by itself could not be used to produce an effective film [70]. However, with the addition of other materials, the weak mechanical properties of sericin can be overcome [71]. It has been reported that the Food and Drug Administration (FDA) has already approved sericin globular protein and its derivatives for inclusion in the generally recognized as safe list (GRAS notice GRN 1026), with no evidence of causing allergy when administered orally and with no cytotoxicity effects as an ingredient of cosmetics, and such applications of silk sericin are apparent in developed countries such as the USA, Japan, Austria, China, Italy, and Romania [1,6]. During recent times, the use of sericin in the food industries is mainly focused use as a food supplement because of its easy availability, nontoxicity, and functional properties [72,73].

The polarity of sericin influences its affinity and the hydrophobicity or hydrophilicity of sericin films [74]. Use of the higher polarity type results in the formation of sericin film with a higher moisture content [74]. Conversely, the addition of sericin hydrolysate to sericin film induces not only a reduction in moisture content but also lower molecular weight, which in turn increases the water-vapor permeability [74]. Moreover, the addition of sericin hydrolysate results in higher antioxidant activity due to the biosynthesis of polyphenols and alkaloids and the total phenolic content than for sericin-only film due to strong acid hydrolysis [74]. 

Nanocelluloses, such as bamboo-derived cellulose nanofibrils, can also be used to strengthen sericin film, thus addressing its insufficient physical qualities [71]. Sericin film combined with bamboo-derived cellulose nanofibrils shows promising antioxidant activity [71]. The incorporation of polysaccharide polymers (glucomannan) combined with glycerol results in the form flexible sericin films [70]. Furthermore, sericin can be used in combination with other biopolymers to minimize the permeability of films by lowering the amount of plasticizer to maintain flexibility [70]. 

Another author has reported that the use of a sericin-based edible coating material containing chitosan, aloe vera, and glycerol has the potential to prolong the storage life of tomatoes under storage at 25 °C and relative humidity of 70% [21]. Compared with uncoated fruits, it is possible to maintain the number of fruits and prevent aging comparably to postharvest conditions [21]. Additionally, ATR–FTIR analysis shows that the coating material does not affect the structure of the fruit [21]. Another study showed that sericin films with elongation characteristics could be obtained by adding glycine as a plasticizer. Glycine demonstrates synergistic actions with water molecules in acting on the sericin film, which increased the elasticity, and as the amount of glycine in the sericin film was increased, the moisture level and β-sheet structure also increased moderately, thereby increasing its elasticity properties [75]. Thus, glycine plasticization increases the moisture level of films [75].

**Figure 5:**
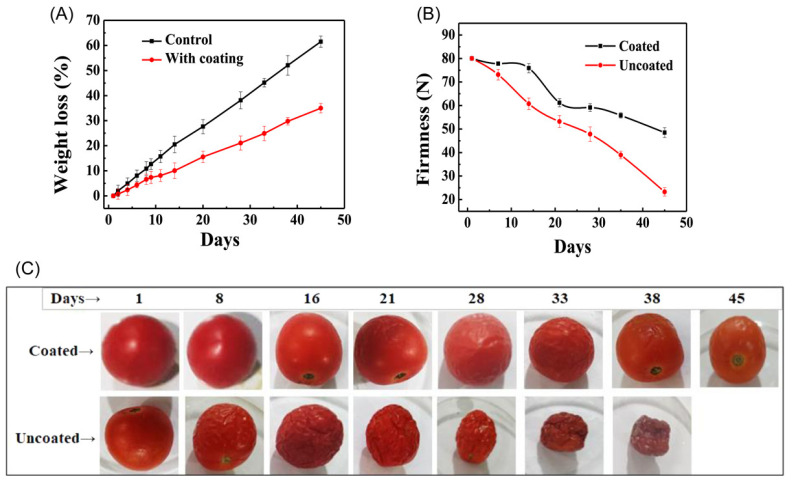
(**A**) Effect of sericin-based edible coating on weight loss; and (**B**) effect of sericin-based edible coating on the firmness for a period of 45 days of storage at 25 °C. (**C**) Photos of the tomatoes with and without the sericin-based edible coating. Reproduced from Tarangini et al. [21], distributed under the terms of the Creative Commons CC BY license, 2022, John Wiley and Sons (originally Figure 1).

In another study, Tarangini et al. studied the properties of sericin-based edible coating materials on the shelf-life and quality of tomatoes during storage for a period of 40 days at 25 °C and relative humidity of 70%, and they concluded that the developed sericin-based coating material was able to decrease the losses in weight and firmness in the tested tomatoes (Figure 5A–C) [21]. Moreover, with the increase in the storage time, the titratable acid content of the samples increased, and the pH, total soluble solid content, total phenol content, total antioxidant, and lycopene content remained low compared to those of the uncoated tomatoes [21].

Another research study by Oh et al. [69] showed the potential for the use of sericin in food packaging. Sericin films with tight crosslinks were employed to fabricate glucose and heat treatment and thus enhance the restricted physicochemical capabilities of sericin by triggering the sericin–glucose Maillard reaction [69]. The author studied the hydrophilicity of the sericin film surface by using the contact angle, which is used to evaluate the hydrophilicity of the polymer surface [69]. The author stated that, with the progression of the chemical crosslinking reaction based on the Maillard reaction, the inherent weaknesses of sericin film, such as the mechanical properties and water resistance, were significantly improved, and it also endowed the sericin film with UV protection and antioxidant properties [69]. Thus, by applying glucose to create a sericin coating, food oxidation can be controlled, and the shelf life can thus be lengthened [69]. 

To supplement the mechanical properties of sericin, a film was prepared by combining it with agarose, one of the neutral polysaccharides [76]. After coating with polydopamine, which has excellent adhesive properties regardless of surface properties, Ag/ZnO nanoparticles were captured to improve the antibacterial activity [77]. As a result, the prepared coating showed excellent antibacterial activity against Gram-negative bacteria *E. coli* and Gram-positive bacteria *S. aureus* [76]. Therefore, the bonding of sericin/agarose film with Ag/ZnO not only improves the mechanical properties of sericin but also results in an excellent antibacterial performance. Films with these characteristics as polymeric materials show potential for use in covering other surfaces to kill microorganisms [76].

Sericin could be used as a potential candidate in the manufacture of natural food-packaging materials [78]. The environmentally friendly process for manufacturing sericin film for its potential applications in food packaging offers both cost-effectiveness and environmental friendliness [71,74]. As a result, sericin protein can also reduce the amount of food waste because it can prolong food storage (https://neo.life/2022/06/food-lasts-1-week-longer-with-this-edible-silk-coating/, accessed on 21 December 2022). 

**Table 4:** Use of sericin in combination with other materials for application in food-packaging industries.

	Limitation	The Supplements Used with Sericin	References
Sericin-based food-packaging materials	The weak mechanical properties promoted the sericin self-aggregation	Nanocellulose can also be used to strengthen a sericin film’s limit regarding its insufficient physical qualities, such as bamboo-derived cellulose nanofibrils.	[71]
		Using sericin in combination with other biopolymers can minimize the permeability of the films by lowering the amount of plasticizer to maintain flexibility.	[70]
		Chemical crosslinking reaction between sericin and glucose helps to overcome sericin film’s limitations, such as water resistance and mechanical properties.	[69]
		Glycine together with sericin generates a synergistic action with the water molecule on the sericin film, thereby increasing the elasticity, moisture level, and β-sheet structure of the sericin film moderately.	[75]
		Sericin/agarose film bonded with Ag/ZnO not only improves the mechanical properties of sericin but also has excellent antibacterial performance.	[76]
	Hydrophilicity-it is delicate in a water environment	Sericin film added with hydrolysate increases the water vapor permeability.	[74]
		Sericin films incorporated with glucomannan have greater solubility combined with glycerol; thus, sericin has a flexible functionality without raising the film’s water vapor permeability.	[70]
	Preservation	The sericin-based edible coating material, including chitosan, aloe vera, and glycerol, has the potential to prolong the storage life of tomatoes under storage at 25 °C and relative humidity of 70%.	[21]
		By applying glucose to create a sericin coating, food oxidation can be controlled and the shelf life lengthened.	[69]

### 4.2. Other Food Applications of Sericin

The food application of sericin has been reported in 56 patents, and a few examples of the interesting applications of sericin in the food industries are as follows: a method for preparation of silkworm sericin drinks (Patent No. CN103126028A) (https://patents.google.com/patent/CN103126028A/en?oq=CN103126028A, accessed on 21 December 2022); inhibitor of bitter or astringent taste for beverages or foods, using a casein phosphopeptide or sericin as an active ingredient (Patent No. JP2012217442) (https://patents.google.com/patent/JP2012217442A/en?oq=JP2012217442, accessed on 21 December 2022); food and drinking water containing sericin and/or sericin hydrolysate (Patent No. JP2000184868) (https://patents.google.com/patent/JP2000184868A/en?oq=JP2000184868, accessed on 21 December 2022); another patent on the utilization of sericin peptide for making silk gum health food (Patent No. CN107080260) (https://patents.google.com/patent/CN107080260A/en?oq=CN107080260, accessed on 21 December 2022); a composition containing sericin with hypotensive action (Patent No. JP2004269395) (https://patents.google.com/patent/JP2004269395A/en?oq=JP2004269395, accessed on 21 December 2022); the use of sericin peptide for rehydrating the rose flower used for making rose-flavored wine (Patent No. CN105524762) (https://patents.google.com/patent/CN105524762A/en?oq=CN105524762, accessed on 21 December 2022); and a patent on using sericin peptide as an ingredient for making a low-sugar probiotic dextrose candy that is suitable for consumption by diabetics (Patent No. CN103918853A) (https://patents.google.com/patent/CN103918853A/en?oq=CN103918853, accessed on 21 December 2022).

**Figure 6:**
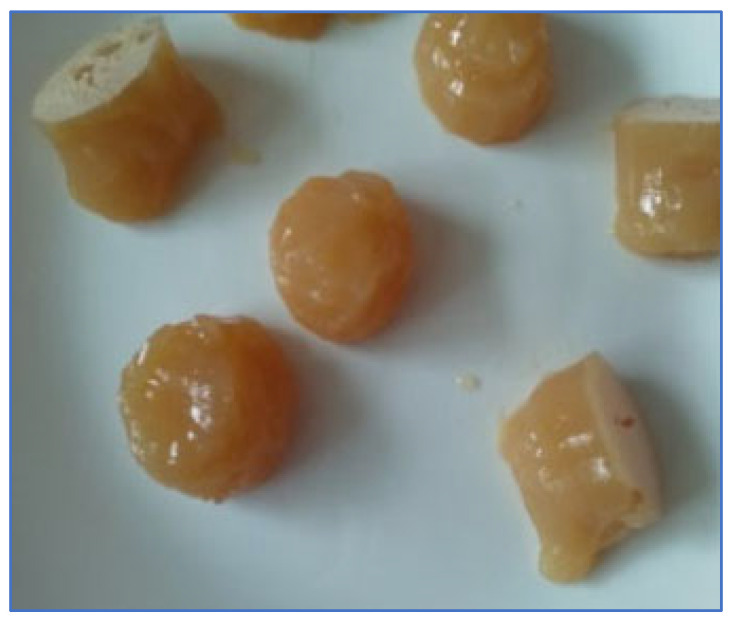
Jellies made with sericin as an ingredient. Reproduced from Matran et al. [67] distributed under the terms and conditions of the Creative Commons Attribution License (CC BY) by MDPI, 2022, Licensee MDPI, Basel, Switzerland (originally Figure 1).

Apart from these, there are several uses of sericin in food-sector industries. Sericin has been reported to be used as an ingredient in bread [6,73]. Sericin was combined in the baking of bread as an endorsement of the beneficial properties of sericin [73]. The author indicated that combining sericin at a calculated dose of 2–4 g of sericin/1 kg of flour for making bread tends to lower the height and specific volume of the bread, along with its color, while sustaining a uniform internal surface texture and taste [73]. Another author reported the use of sericin as an ingredient in salad dressing [79]. The author stated that sericin with no immunogenicity potential [80] can be preferably used as an emulsifying agent over natural food emulsifiers such as egg yolk and casein, which sometimes have a potential risk of allergic reactions [79]. Furthermore, the emulsifying activity of sericin can be enhanced by acylation with oleic acid [81]. Similarly, another author reported the use of sericin in making jelly deserts [67], wherein a sustainable jelly was prepared using depectinized apple juice, pectin, sericin, lactoferrin, stevia, and pectin (Table 5, Figure 6). The author stated that the sericin glycoprotein could be used to obtain jelling foods with low energy value, including foods for patients with dysphagia [67].

By observing the hardening phenomenon of the high-protein nutrient bar to which sericin peptide was added, sericin could improve the mobility of water and small hydrophilic molecules in the sample, thus lowering the phase separation rate. After the addition of the sericin peptide, the ζ-potential, secondary structure content, and surface hydrophobicity of the sample were also changed to prevent self-aggregation of the protein. These results indicate that sericin reduces the curing phenomenon of the samples and can be used as a promising anti-curing ingredient in the food industry to improve the texture of food [82,83].

There are some reports on improvement of the storage stability of fruits and vegetables by the application of sericin [84]. Furthermore, when it is used as a food component, sericin significantly impacts the texture of food by enhancing its compatibility, mechanical potential, and the shape and size of the food product; for example, it can decrease the hardness and improve the elasticity of steamed potato bread according to Gong et al. [85].

### 4.3. Challenges and Benefits of Using Silk Sericin Protein Materials in Food Products

Apart from its health-benefiting potential, sericin has a few limitations that make it unsuitable for potential application in food-sector industries. This applies also to its use in food packaging, which is limited by its feeble mechanical properties as a result of the self-aggregation process of sericin, which is responsible for the weak mechanical strength [56]. Moreover, its hydrophilic nature makes it inappropriate for use in a water environment, and this is an important shortcoming that precludes its use in a variety of industries [57]. Furthermore, the limited solubility of the powder sericin in solvent and the unpleasant smell of silkworm chrysalises are barriers to its unrestrained use in healthy and functional foods and additives [86]. Considering this, modification of the properties of sericin concerning its solubility in water and the removal of unfavorable flavors is an important task for researchers. The application of Maillard reaction techniques in food-related proteins is reportedly effective in increasing the functional properties of a food item, which exhibits subsequently enhanced emulsifying and antioxidant properties [87,88,89,90]. Furthermore, some researchers have also used the glycation method to enhance the application of sericin to functional foods [69]. Wang and Zhang reported that the solubility in water is increased 2–3 times when sericin is glycated, and its antioxidant capacity increases 3.5–5.5 times regardless of the kind of sericin and reducing sugar used [86]. Moreover, the pupal scent of sericin is also specifically removed by glycation and is replaced with a subtly sweet, mellow scent [86]. Therefore, the glycation technique has been proven to have a variety of application opportunities for sericin as a functional food, health food, or food additive, as well as for promoting the sustainable growth of the silk industry [86].

An allergic reaction to food occurs when the body’s immune system recognizes some proteins in food as foreign and defends against them. Adverse effects on these foods are caused by protein additives [91]. It is reported that sericin, being a natural biomaterial, is safe for biological systems, with low immunogenicity and eliciting almost no allergic responses [92].

## 5. Other Potential Applications of Sericin

Besides its application in food-sector industries, sericin has numerous other applications, some of which are as follows (Table 6).

### 5.1. As an Antibacterial Agent

Sericin has been reported to possess antibacterial activity; however, there is a difference in the antibacterial activity depending on the various degumming methods used for extraction of sericin. The higher the density of sericin extracted by sodium carbonate in the degumming process, the greater its antibacterial activity [33]. Regarding its antibacterial activity, sericin extracted using a degumming process involving sodium carbonate demonstrated inhibition against foodborne pathogenic *E. coli* bacteria [93]. Sericin obtained by a water degumming method shows potential inhibition of foodborne pathogenic *S. aureus* bacteria [93]. In another report, sericin demonstrated a potential inhibition effect against both Gram-negative and Gram-positive bacteria based on optical density (OD_600nm_) measurements [94]. 

Chemicals or enzymes in silk cocoons containing sericin may act against invaders such as microbes [95]. This is most likely because several amphipathic fundamental polypeptides lyse the microorganisms, resulting in their death [95]. Zhang et al. reported that peptides of sericin under 29 kDa have antibacterial potential when combined with other biopolymers, such as chitosan nanofibers; or film or chemical agents, such as silver nanoparticles, zinc oxide nanoparticles, antibiofilm titanium, etc. [96]. These factors explain how purity and extraction methods impact sericin’s antibacterial activity [97]. It has been reported that, upon degumming, the antibacterial activity improved, and there was a decreased loss of sericin [56]. This shows that the higher the concentration of sericin, the better the antibacterial properties. Higher crosslinking at pH 7.0–8.5 and temperatures of 30–40 °C gave relatively good sericin fixing-effects [56].

### 5.2. Antioxidant Potential of Sericin

Although reactive oxygen species (ROS) are generated throughout cellular metabolism, they become toxic at high concentrations [98]. ROS with free radicals are highly reactive with other substances due to their instability [98]. In the food industry, synthetic antioxidants such as BHT and BHA have normally been used, as they are more potent and affordable than natural antioxidants [98]. However, this is always accompanied by anxiety about using synthetic antioxidants because of health safety [98]. Therefore, natural protein sources are preferred instead of synthetic antioxidants [98]. Sericin, as a natural macromolecular protein, has functional groups such as Tyr, Trp, His, and Cys [98]. Due to their propensity to donate hydrogen and interact, they produce more stable molecules, using free radicals, and interrupt the radical chain reaction, and functional groups in amino acids reduce and decolorize DPPH [44]. 

**Figure 7:**
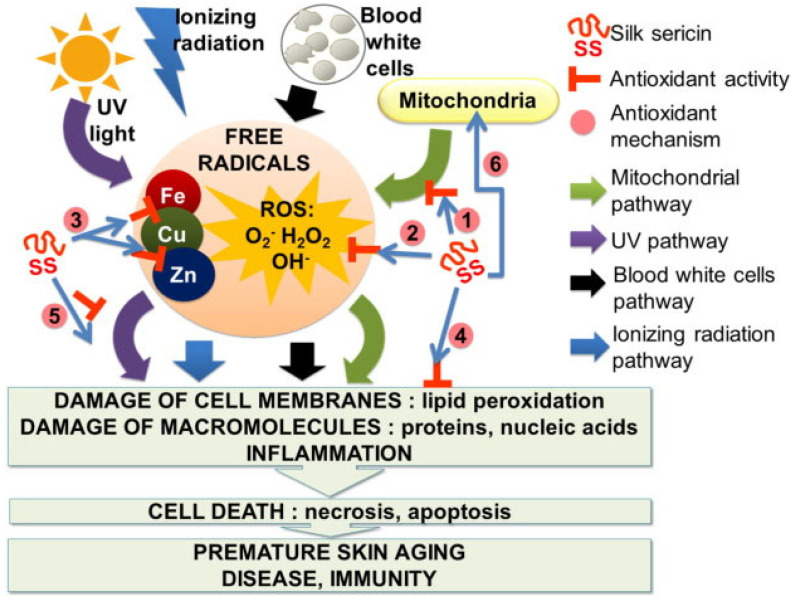
Mode of sericin antioxidant activity. ROS = reactive oxygen species; 1 = enrichment of antioxidant enzymes, i.e., superoxide dismutase and glutathione peroxidase; 2 = ROS scavenging; 3 = chelation of transition metals; 4 = prevention of lipids peroxidation and inflammation; 5 = inhibition of UV-induced apoptosis and sunburn effects on the skin; 6 = increased level of NAD+ and improved detoxification via oxidation. Reproduced with permission from Lamboni et al. [3], 2015, Elsevier (originally Figure 4).

The antioxidant action of sericin hydroxyl groups, such as alcohol, may be due to their capacity to chelate trace metals such as copper and iron, which could partially reverse the effects of oxidative stress on mice liver [35,99]. Free radicals, which cause oxidative damage, are stabilized by the scavenging action of sericin [100]. The molecular size of sericin is inversely related to its antioxidant activity. Its molecular size can thus be reduced to increase its antioxidant activity [101]. In addition, low-molecular-weight sericin peptides have demonstrated an outstanding scavenging activity ability [43]. Furthermore, it is noteworthy that sericin’s antioxidant action is caused by the presence of polyphenols and flavonoids [18]. Consequently, it has been discovered that the hydrogen-donating properties of amino acids, hydroxyl groups, high-molecular-weight sericin, polyphenols, and flavonoids allow the diminishing and decolorizing of DPPH [98]. A possible mode of antioxidant potential of sericin was discussed by Lamboni et al. [3] (Figure 7). Therefore, sericin is a valuable multifunction substance that can be explored for its application in cosmetics and food-preservative industries as a natural and safe ingredient with respect to the oxidative processes that influence food quality and shelf life [12,18,100].

### 5.3. Anticancer Potential of Sericin

Sericin protein has been demonstrated to have potential anticancer activity against numerous types of cancer cells, in addition to its antioxidant effects [94]. Various studies have shown the anticancer effects in such models as breast, colon, colorectal, lung, cervical, and prostate cancer cells [102]. Cancer cells treated with sericin show decreasing viability with increasing concentrations of sericin [102]. Silk sericin based self-assembled nanoparticles stimulates apoptosis in the MCF-7 breast cancer cells [103]. This indicates that sericin has more toxic effects on cancer cells, such as MCF-7 cells in breast cancer [102]. Moreover, it was found to significantly suppress the proliferation of triple-negative breast cancer (TNBC), which is known to be difficult to treat and has a poor prognosis, and cell apoptosis is promoted via the inhibition of signaling pathways such as PI3K/Akt [104]. The anticancer effects of sericin were found to be due to a reduction in Bcl-2, which is a regulatory factor that promotes or inhibits apoptosis, and increased activity of caspase-3, which is an active factor in the end process of apoptosis that stimulates the apoptosis of colon cancer cells [94]. Sericin-based material has been reported to be highly effective as an apoptosis agent against cancer cells such as of breast, colon, lung, and liver cancer cells [94]. Furthermore, it shows a reduction effect on 1,2-dimethylhydrazine agents, which are cancer-growth promoters. The incidence of colorectal cancer is decreased with the mitigation of such agents [1]. 

Kaewkorn et al. reported the protective effect of silk sericin against colon cancer cells and suggested that sericin might have substantial health-benefiting effects and could be established as a dietary supplement for preventing colon cancer [105]. Likewise, sericin has demonstrated properties of cancer inhibition, such as of human lung, cervical, and prostate cancer, by inducing apoptosis and cell cycle arrest [106]. The anticancer action of sericin shows that it affects cancer cells by causing morphological changes, inciting cell shrinkage, and inducing nuclear condensation [94]. Accordingly, sericin has anticancer properties, such as activating apoptotic pathways, and may thereby contribute to the prevention of angiogenesis, suspension of the tumor cell cycle, and control of cancer progression [94].

### 5.4. Anti-Tyrosinase Potential of Sericin

Sericin has been reported to possess anti-tyrosinase properties with suppression of polyphenol oxidase [15,35]. The flavonoids and carotenoids that accumulate in sericin layers are responsible for endowing sericin with anti-tyrosinase activity [14,18]. Wu et al. demonstrated that sericin has the potential to strongly inhibit tyrosinase [39]. Various methods for extraction of sericin have been reported to have a role in the effective anti-tyrosinase potential of sericin, and the anti-tyrosinase activity is highest when it is extracted with urea [55]. Large amounts of arginine and valine, which are bound to tyrosinase enzyme, are extracted in high amounts during the process of sericin extraction with urea, which might thus increase the anti-tyrosinase activity of sericin [100]. Moreover, tyrosine can promote oxidative activity as an enzyme substrate [100]. Sericin biopeptides demonstrate anti-tyrosinase effects owing to numerous serine, asparagine, and threonine residues acting as chelators to present the most inhibitory effects [100]. 

Anti-tyrosinase activity is increased when copper ions of tyrosinase enzyme are active because they are bound by sericin [100]. On the contrary, hydrophobic amino acids such as valine, alanine, and leucine have the anti-browning properties of sericin hydrolysate due to the metal-chelating abilities of amino acids because they are bounded by hydrophobic pockets near the tyrosinase enzyme [84]. Apart from serine and valine, arginine enhances the anti-tyrosinase activity of sericin because it can bind and inhibit tyrosinase enzymes [107]. The anti-tyrosinase activity is known to relieve diseases such as Parkinson’s and cancer, since it prevents the production of melanin, which results in the browning of many fruits and vegetables [100]. Therefore, it has wide potential in food and biomedical applications [100].

### 5.5. Anti-Inflammatory Potential of Sericin

The covalent interactions of sericin have a variety of effects on its anti-inflammatory properties, and synergistic effects with several distinctive compounds have been reported [108]. Silk sericin grafted with phenolic compounds such as pyrogallol and hydroquinone has significant anti-inflammatory properties [108]. An important factor in the etiology of inflammation is nitric oxide, and if the NO content increases, inflammation is enhanced [108]. Moreover, 15-lipoxygenase (15-LOX) helps convert arachidonic acid to leukotriene, and it is an important pro-inflammatory lipid mediator that is related to inflammatory diseases [108]. The anti-inflammatory activity of control sericin (CS), pyrogallol–sericin conjugate (PS), and hydroquinone–sericin conjugate (HS) was assessed by NO production and 15-lipoxygenase (15-LOX) inhibitory assays [108], with the conclusion that PS conjugates are more potent than CS and HS, as indicated by remarkable 15-LOX inhibition activity compared with the positive control quercetin [108]. 

Another study showed that inducible nitric oxide synthase (iNOS) and cyclooxygenase-2 (COX-2) genes attributed to inflammatory gene expression are dose-dependently downregulated by treatment with sericin [109]. Moreover, silk sericin not only inhibits in vivo carrageenan-induced inflammation due to the activation of anti-inflammatory reactions but also regulates the production of pro-inflammatory cytokines [109]. 

Gel formulations containing both silk sericin obtained from a natural source and alginate may be used in the treatment of inflammation because they are more effective than gels formulated only containing silk sericin in suppressing carrageenan-induced inflammation [110]. The combination of silk sericin with naringin results in diminished mRNA expression and production of pro-inflammatory cytokines in human endothelial cells (TNF-α, IL-6, IL-12p40, and IL-23) (hPBMCs) [111]. In another study, rat tissue treated with both indomethacin, an nonsteroidal anti-inflammatory agent, and silk sericin presented similar numbers of macrophage cells and polymorph nuclear cells [109]. These results suggest that sericin in combination with other biomaterials could be used purposefully in various medical applications. 

### 5.6. Anti-Aging Potential of Sericin

A powder made of sericin (5–30%) and silk fibroin (70–95%) exhibits antistatic and moisture-absorbing capabilities [112]. Silk sericin has high water solubility (hydrophilic features), which is an important factor in cosmetics [113]. Sericin with low molecular weight acts as a glue and is well-known for its use in cosmetics for skin, hair, and nails, because it improves the elasticity of skin and has antiaging and anti-wrinkle effects [112,114]. The abilities of silk sericin to retain moisture, gel, and promote skin adherence are its primary cosmetic characteristics [113]. Sericin inhibits apoptosis and stimulates collagen type I synthesis. In addition, it surpasses vitamin C, which has effective anti-aging properties, by limiting oxidative stress [115]. Its anti-apoptotic effects were also investigated in human epidermal keratinocyte cells irradiated with UVB (30 mJ/cm^2^) [114]. It inhibits tyrosinase activity and intracellular hydrogen peroxide generation and regulates nitrite, which causes oxidative stress, and the development of b-cell lymphoma 2 is increased (bcl-2) [114,115]. These findings imply that sericin may be able to mitigate mitochondrial damage [114]. Gels made with silk sericin containing pluronic and polyacrylic acid prevent trans-epidermal water loss from the skin because skin impedance is decreased and hydroxyproline content in skin and hydration of epidermal cells are increased [112,114]. Therefore, it can restore natural moisturizing factors and stimulate moisturizing properties [112]. Silk sericin peptide fractions also have potential anti-aging properties [114]. According to the results, silk sericin possesses anti-aging properties that could be successfully exploited in cosmeceuticals, namely lotions, creams, and ointments [112,113,115]. In addition, it can be used to control skin conditions such as dermatitis [112].

### 5.7. Other Potentials of Sericin

The effect of dietary sericin protein on the skin dryness of atopic dermatitis (AD) with poor epidermal hydration was investigated [116]. Epidermal hydration is primarily maintained by natural moisturizing factors, one of the components of which is produced by the breakdown of filaggrins, a free amino acid. Dietary sericin improves epidermal hydration in parallel with degradation to free amino acids coupled with elevated levels of peroxisome proliferator-activated receptor gamma, peptidylarginine deiminase-3, and caspase-14 proteins and enhanced profilaggrin expression [116].

The effect of dietary sericin protein on the skin dryness of atopic dermatitis (AD) with poor epidermal hydration was investigated [116]. Epidermal hydration is maintained primarily by a natural moisturizing factor, one of the components of which is produced by the breakdown of filaggrins, which are a type of free amino acid. Dietary sericin improved epidermal hydration in parallel with degradation to free amino acids coupled with elevated levels of peroxisome-proliferator-activated receptor gamma, peptidylarginine deiminase-3, and caspase-14 proteins and enhanced profilaggrin expression [116].

**Table 6:** Biological properties of sericin.

Biological Properties	Target	Influence	References
Antibacterial activity	- Sericin promotes the blebbing of bacterial cell membranes, thus preventing the growth and reproduction of bacteria.	Sericin purity and extraction process.	[97]
	- *Micrococcus luteus* exhibits its antibacterial characteristics through its impregnated layers.	Sodium carbonate in the density of 10 mg/mL.	[32]
	- Sericin inhibits the growth of *E. coli* and *S. aureus.*	- Sericin extracted for 90 min in hot-water methods at 30 μg; - Sericin extracted for 60 min in Na_2_CO_3_ methods at 40 μg.	[93]
	- Pathogen-specific response of the polypeptide (*E. coli*, *B. cereus*, *S. aureus*, *K. pneumoniae*, and *P. aeruginosa*) may be due to phagocytosis.	The extracted sericin using physical methods at 50 mg.	[95]
	- Sericin suppresses bacteria’s metabolism, formation of cell walls, peptides that bind to lipopolysaccharides, and protein folding (*E. coli*, *S. aureus*, *Vibrio cholera*, *Salmonella typhi*, and *Shigella flexneri*).	Sericin was extracted using sodium chloride and ethanol at different concentrations (25, 50, 75, and 100 mL).	[94]
	- Sericin has the potential for antibacterial activity due to its combination with other antibacterial bioactive molecules.	The peptide of Sericin under 29 kDa.	[96]
Antioxidant activity	- Alanine and glycine have intracellular antioxidant properties; - Mitochondrial structural preservation through the control of prohibitin-2, mitochondrial elongation factor Tu, and NADH-ubiquinone oxidoreductase, intracellular proteins that control apoptosis and autophagy.	Its purity and extraction process.	[12]
	- Sericin can protect against oxidative stress and decrease reactive oxygen species.	Its purity and extraction process.	[98]
	- By using the DPPH and ABTs assay to diminish the size of sericin, its antioxidant activity might be increased.	The extraction time has changed (15, 30, 60, 90, and 120 min),	[101]
	- Sericin might be the prevention of a chain reaction carried on by free radicals.	Male mice were given at different concentrations (0.375, 0.75, and 1.50 g/kg b.w.).	[99]
	- Sericin has an antioxidant activity that reacts with free radicals to turn them into more stable molecules and stop the chain reaction from starting.	Using samples of pure sericin (A1) and crude sericin extracts (A2 and A3) at concentrations ranging from 5 to 60 mg/mL.	[44]
	- The scavenger activity increased as a result of its reduction in size.	The bacterial-purified protease.	[100]
	- The antioxidant defense system against ROS elements includes glutathione (GSH), superoxide dismutase (SOD), catalase (CAT), and glutathione peroxidase (GPx).	Various extraction methods (conventional, autoclaving, urea degradation, alkali degradation, and acid degradation) and silk-cocoon varieties.	[43]
	- Sericin substantially decreases intracellular ROS shown on fluorescence; - It has been hypothesized that sericin’s primary amino acids protect *B. mori*’s hemocytes and midgut epithelial cells against oxidative damage, most likely because of sericin’s ability to scavenge ROS; - The polyphenols and flavonoids in sericin are what give it its antioxidant qualities.	Its purity and extraction process.	[18]
	- Sericin’s high serine and threonine concentrations, as well as its 40% hydroxyl content, act as antioxidants by chelating trace metals such as copper and iron.	Amounts of 0.0%, 0.5%, and 1.0% sericin.	[35]
	- 10 μg/mL of sericin from Bombyx mori shows 50% of DPPH radical scavenging; - When the sericin concentration increased, the amount of radical scavenging activity also did in a dose-dependent manner.	Amounts of 10, 20, 40, 80, and 100 μg/mL of silk sericin.	[117]
Anticancer activity	- Silk sericin based self-assembled nanoparticles stimulates apoptosis in the MCF-7 breast cancer cells.	The method of sericin isolation and the families of the silk cocoon.	[103]
	- Cancer cells, such as breast, colon, colorectal, lung, cervical, and prostate cancer cells, treated with sericin show decreasing viability owing to increasing the concentration of sericin.	Sericin from *Antheraea assamensis*, *Bombyx mori*, and *Philosamia ricini* was measured at 0.5, 1.0, 2.0, and 4.0 mg/mL.	[102]
	- The reduction of Bcl-2, which is a regulatory factor that promotes or inhibits apoptosis, and increased activity of caspase-3, which is an active factor in the end process of apoptosis, stimulate the apoptosis of colon cancer cells; - Sericin damages cancer cells, causes morphological changes, encourages cell shrinkage, and induces nuclear condensation.	Amounts of 0, 0.25, 0.50, 0.75, and 1.0 mg/mL of sericin.	[94]
	- Sericin shows the reduction effect of the 1,2-dimethylhydrazine agents, which is a cancer-growth promoter.	Sericin hydrolysis by using chemical, thermal, or physical extraction processes.	[1]
	- Sericin significantly suppressed the proliferation of triple-negative breast cancer (TNBC), which is known to be difficult to treat and has a severe prognosis and advanced cell apoptosis by inhibiting the signaling pathway, such as PI3K/Akt.	Amounts of 0, 0.5, 1.0, 2.0, 4.0, 8.0, and 16.0 mg/mL of sericin.	[104]
	- Sericin is considerately effective on SW480 cells, which are isolated from colorectal cancer as the inhibitory since it seems to decrease their viability and induce apoptosis.	Sericin of various sizes and concentrations (ranging from 25 to 1600 mg/mL)	[105]
	- Sericin has properties for cancer inhibitory, such as human lung, cervical, and prostate cancer, by inducing apoptosis and cell-cycle arrest.	Amounts of 0.2, 0.3, 1.0, 1.5, 3.0, 5.0, and 10.0 μg/μL of sericin.	[106]
Anti-tyrosinase activity	- The flavonoids and carotenoids which are accumulated in sericin layers were caused to endow sericin with anti-tyrosinase activity.	Its purity and extraction process.	[14,18]
	- Sericin inhibits the tyrosinase enzyme.	Basin, deflossing, and reeling waste of sericin.	[19]
	- Sericin has anti-tyrosinase properties of various biological functions that suppress polyphenol oxidase.	Amounts of 0%, 0.5%, and 1.0% of sericin	[15,35]
	- Sericin has extracted the highest value of an anti-tyrosinase activity is sericin with urea; - The large amounts of arginine and valine, which are bound with tyrosinase enzyme extracted from the extraction process with urea, could increase anti-tyrosinase activity.	- Sericin from various extraction methods (heat, urea, acid, and base); - Enzymatic digestion, denaturing agents (boiling with detergents, alkaline chemicals, and chaotropic agents), polymeric membranes, infrared radiation, steam, ultrafiltration, and nanofiltration (autoclave).	[55,100]
	- Sericin has the potential to inhibit fruit and vegetable enzymatic browning.	An 8% (*w*/*v*) sericin solution.	[39]
	- Arginine enhances the anti-tyrosinase activity of sericin because it could be bounded and inhibited with tyrosinase enzymes; - Hydrophobic amino acids, such as valine, alanine, and leucine, have the anti-browning properties of SH due to the metal-chelating abilities of amino acids because they are bounded with hydrophobic pockets near the tyrosinase enzyme.	Amounts of 5, 10, and 20 μg/mL of sericin.	[84,107]
Anti-inflammatory activity	- Inducible Nitric Oxide Synthase (iNOS) and Cyclooxygenase-2 (COX-2) genes attributed to inflammatory gene expression are down-regulated by treatment with sericin dose-dependently; - Silk sericin in vivo not only inhibits carrageenan-induced inflammation due to the activation of anti-inflammatory reactions but also regulates the production of cytokines as pro-inflammatory.	Amounts of 0.25, 0.5, 1.0, and 2.0 mg/mL of sericin.	[109]
	- Gel formulations containing silk sericin obtained from both a natural source and alginate may be the treatment of inflammation in suppressing carrageenan-induced inflammation.	Amounts of 20% and 80% sericin-loaded alginate nanoparticle gel.	[110]
	- Expression of mRNA by human peripheral blood mononuclear cells and production of pro-inflammatory cytokines (TNF-α, IL-6, IL-23, and IL-12p40) were reduced when silk sericin and naringin were combined.	Naringin (20 g/mL) and sericin (100 g/mL) combined in a 1:1 ratio (*v*:*v*).	[111]
	- Silk sericin grafted with phenolic compounds such as hydroquinone and pyrogallol has significant anti-inflammatory properties; - Sericin-phenolic conjugates were effective to inhibit 15-LOX activity.	The supernatant was mixed with hydroquinone and pyrogallol in a 1:10 (phenol/sericin) ratio.	[108]
Anti-aging activity	- Sericin inhibits apoptosis and stimulates collagen type I synthesis; - Sericin regulates nitrite, which causes oxidative stress and the development of b-cell lymphoma 2 (bcl-2), is increased.	- 500 μg/mL of sericin; - Days 1, 3, 5, 7, 9, and 11.	[115]
	-Sericin can restore natural moisturizing factors and stimulate moisturizing properties.	Sericin hydrolysis by using chemical, thermal, or physical extraction processes.	[112]
	- Sericin inhibits apoptosis in UVB (30 mJ/cm^2^)-irradiated human epidermal keratinocyte cells and prevent the activation of caspase-3.	Sericin hydrolysis by using chemical, thermal, or physical extraction processes.	[114]
	- In the sericin group, five animals (83.33%) had significant collagen deposition, fibrosis, and fibroblastic activity.	Wistar albino adult male rats weighing between 257 to 395 g were given 30 mg of sericin powder through their thoraxes at the age of 12 weeks.	[113]

## 6. Conclusions and Future Prospects

Silk sericin, which is naturally sourced, is a high-molecular-weight protein produced by the silkworm insect *B. mori.* As a water-soluble glycoprotein, this protein comprises up to 25–30% of a silk cocoon. The mechanical and biological properties of sericin are influenced depending on the type of extraction, and it can be characterized by a wide molecular-weight range of 10–400 kDa, from low to high molecular weight. It also has hydrophobic or hydrophilic amino acids, which can explain its antibacterial, antioxidant, anticancer, anti-tyrosinase, anti-inflammatory, and anti-aging properties. Several applications of sericin, along with its combination with other biomaterials, have been reported, with potential application in food-sector industries. When sericin protein was used in food, it was reported to show very few side effects and allergies; hence, it can be explored further in various aspects as a functional food, food additive, and many more. Moreover, the developed sericin-based coatings/films are demonstrated to be highly effective in the storage of fruits and vegetables. Hence, sericin-based coating materials can be further developed with future potential application in the postharvest storage and preservation of fruits and vegetables, and this could be helpful in solving the most distinct issue of food-sector industries, i.e., food spoilage and degradation.

## Figures and Tables

**Table 1:** Different methods of extraction of sericin, their advantages, and their limitations.

Methods		Advantages	Limitations	Reference(s)
Physical	Hot water	- It is to use natural polymers.	- The purity of sericin was low; - The limited information about the composition.	[29,30]
Chemical	Alkaline solution: Na_2_CO_3_ (dialysis sericin solution containing alkaline or acidic solutions)	- It is low-cost to use on a large scale; - It has good high yield and antioxidant activity.	- Separate ions and molecules in the sericin solution; - Exchange with ions and other molecules.	[3,31,32]
	Alkaline property: Marseille soap	- The soap is a natural material.	- Marseille soap separation is very difficult; - The soap is very expensive.	[3,33]
	CaCl_2_ (Calcium chloride)	- Recover the high-purity sericin from the silk cocoon; - Decrease the amount of microbic waste.	- The dialysis process is essential.	[29]
Enzymatic	A protease enzyme	- DPPH radical-scavenging activity and a three-fold higher total essential amino acid value than the water-extraction method.	- It is expensive to use in large quantities.	[33]

**Table 2:** The amino acid profile of sericin as per different works published in the literature.

Alanine	Arginine	Aspartic acid	Glutamic acid	Glycine	Histidine	Isoleucine	Leucine	Lysine	Methionine	Phenylalanine	Serine	Threonine	Tryptophan	Tyrosine	Proline	Valine	Reference
6.47	9.75	3.82	6.90	12.24	3.50	4.23	2.85	4.76	1.56	7.89	7.01	7.24	6.36	8.58	NA	6.88	[31]
5.71	4.45	NA	NA	12.17	0	1.48	2.03	0.6	0	NA	32.55	7.48	NA	3.69	2.38	6.31	[29]
3.28	4.71	11.52	2.92	12.6	2.05	0.34	1.05	2.33	0.13	0.53	40.51	8.45	NA	5.42	0.59	3.56	[19]
4.1	2.87	15.64	4.61	15.03	NA	0.56	1	2.35	3.39	0.28	33.63	8.16	NA	3.45	0.54	2.88	[34]
5.3	1.8	18	4.6	15.7	1.3	0.7	1.1	2.5	<0.05	0.4	32.2	8.4	NA	3.7	0.6	3.6	[18]
3.8	3.9	17.8	4.4	19.1	1	0.4	0.8	2.7	<0.05	0.2	31	8	NA	3.3	0.4	3.1	[35]
NA	5~15	7~10	4~6	10~20	3~6	NA	NA	20~30	NA	NA	7~16	3~10	NA	4~6	NA	NA	[24]
4.6	2.8	19.1	4.1	12.2	0.9	1.4	0.6	10.2	<0.05	0.4	30.4	6	NA	3.8	0.8	2.6	[37]
6	3.1	16.7	4.4	13.5	1.3	0.7	1.1	3.3	0.04	0.5	33.4	9.7	0.2	2.6	0.7	2.8	[38]
4.6	2.8	19.1	4.1	12.2	0.9	1.4	0.6	10.2	<0.05	0.4	30.4	6	NA	3.8	0.8	2.6	[36]
4.3	4.9	18.8	7.2	10.7	1.7	1.3	1.7	2.1	0.5	1.6	27.3	7.5	0.4	NA	1.2	3.8	[39]
NA	11.95	14	3.3	23.2	1.13	0.91	2.08	3.18	0.77	1.29	21.56	7.04	NA	6.23	NA	3.36	[40]

Values are shown in Mol (%). NA = not available data.

**Table 3:** Variation in the amino acid composition of sericin with respect to different extraction methods.

Amino Acid	Extraction Method of SS			
	Heat	Urea	Acid	Alkaline
Asp	15.64	18.31	15.93	19.88
Ser	33.63	31.27	31.86	30.01
Glu	4.61	5.27	5.75	5.93
Gly	15.03	11.23	10.49	11.01
His	1.06	3.26	2.47	1.72
Arg	2.87	5.41	4.92	4.92
Thr	8.16	8.36	8.51	6.49
Ala	4.10	4.33	3.72	4.21
Pro	0.54	1.46	0.78	1.24
Cys	0.54	0.39	0.53	0.23
Tyr	3.45	0.36	5.56	5.24
Val	2.88	2.96	2.95	2.94
Met	3.39	0.12	0.06	0.15
Lys	2.35	3.14	3.48	2.89
Ile	0.56	0.96	0.87	0.75
Leu	1.00	1.58	1.43	1.56
Phe	0.28	0.60	0.71	0.81

Reproduced under the terms and conditions of the Creative Commons Attribution license (http://creativecommons.org/licenses/by/3.0/) from Aramwit et al. [34], 2010, licensee Molecular Diversity Preservation International, Basel, Switzerland (originally Table 2).

**Table 5:** Sericin used for making desserts.

The Raw Material	Identification Data	How to Get It
Sericin	Batch: S1911251	From the supplier ^1^
Bovine lactoferrin	Batch: 107CLXP	From the supplier ^2^
Chicory inulin ^3^	Batch: RHBGD1BGD1	Purchased from local trade ^4^
Citrus pectin	Batch: 5999884818779	Purchased from local trade ^4^
Stevia	Batch: 8	Purchased from local trade ^5^
Apple juice (depectinized)	Batch: Bm 095-22	Purchased from local trade ^6^
Agar–agar	Batch: 210040401	Purchased from local trade ^7^
Lemon	-	Purchased from local trade

^1^ Sollice Biotech, Toulouse, France; ^2^ KUK, Tunari, Romania; ^3^ cicory—Cichorium intybus; ^4^ importer/distributor: Adams Vision SRL Targu Mures, Târgu Mureș, Romania; ^5^ packaged by Sly Nutritia SRL, Buzau, Romania; ^6^ manufacturer S.C. Pombis S.A., Bistrita, Romania; ^7^ manufacturer Biovegan GmbH, Bonefeld, Germany. The data are reproduced from Matran et al. [67] and distributed under the terms and conditions of the Creative Commons Attribution License (CC BY) by MDPI, 2022, Licensee MDPI, Basel, Switzerland (originally Table 1).
